# Open Reduction and Internal Fixation: An Alternative Approach to Excision of Symptomatic Bipartite Patella

**DOI:** 10.7759/cureus.78819

**Published:** 2025-02-10

**Authors:** Douglas McHale, Christopher Riveria-Pintado, Daniel R Baka, Matthew T Kleiner

**Affiliations:** 1 Orthopaedics, Cooper Medical School of Rowan University, Camden, USA; 2 Orthopaedics, Cooper University Hospital, Camden, USA

**Keywords:** open reduction internal fixation, orthopedic sports medicine, pediatric case, pediatric sports injury, symptomatic bipartite patella

## Abstract

A 16-year-old patient with no relevant medical history presented with pain superolateral to the patella after a sports-related injury. Subsequent imaging revealed an anatomic bipartite patella with a disrupted fibrocartilaginous junction.

Anatomic bipartite patella are normal variants that typically cause minimal to no pain. Painful variants are most often corrected with surgical excision of the excess bone. Open reduction and internal fixation (ORIF) of the accessory bone to the patella is a rare but proven strategy to reduce pain without excision, especially in patients with tendinous attachment to the accessory bone.

## Introduction

The patella is the largest sesamoid bone in the human body. Sesamoid bones are bones that form within a tendon. The patella begins the ossification process from cartilage to bone between the ages of 3 and 5 until 9 to 10 [1]. While more than 75% of people have a single ossification center, the other 25% of the population has more than one [1]. In the case of a bipartite patella, two ossification centers do not fuse, resulting in a fibrocartilaginous junction - an area where two parts of the patella are connected by cartilage and fibrous tissue between the two fragments of bone [2].

The most commonly utilized classification system for bipartite patellae is the Saupe Classification. This classification system is based on the location of the accessory bone. There are three types of bipartite patella: Type I is located at the inferior pole of the patella, Type II at the lateral margin, and Type III at the superolateral pole. The prevalence of each type is as follows: Type I is present in about 5% of patients, Type II in about 20% of patients, and Type III in about 75% of patients [3]. These anatomical variants are usually asymptomatic until there is a direct trauma to the fibrocartilaginous junction, leading to localized edema, pain, and possible instability [4]. The majority of cases associated with disruption of the synchondrosis between the main and accessory portions of a bipartite patella can be managed conservatively with ice, over-the-counter pain medications, bracing, and rest. If conservative therapies fail for a patient, the most common surgical procedure is an excision of the accessory bone. This case report aims to illustrate the surgical treatment of a symptomatic bipartite patella through open reduction internal fixation as a viable alternative to the excision of accessory bone.

## Case presentation

A 16-year-old male, who is 70 inches (1.78 m) tall and weighs 232 pounds (105.2 kg) with a BMI of 33.3 kg/m², presented with several months of superolateral patella pain following a fall during a basketball game. He also endorsed symptoms of instability, which improved with the use of a brace, pain that worsened with exertion, and minimal relief from nonsteroidal anti-inflammatory drugs.

**Figure 1:**
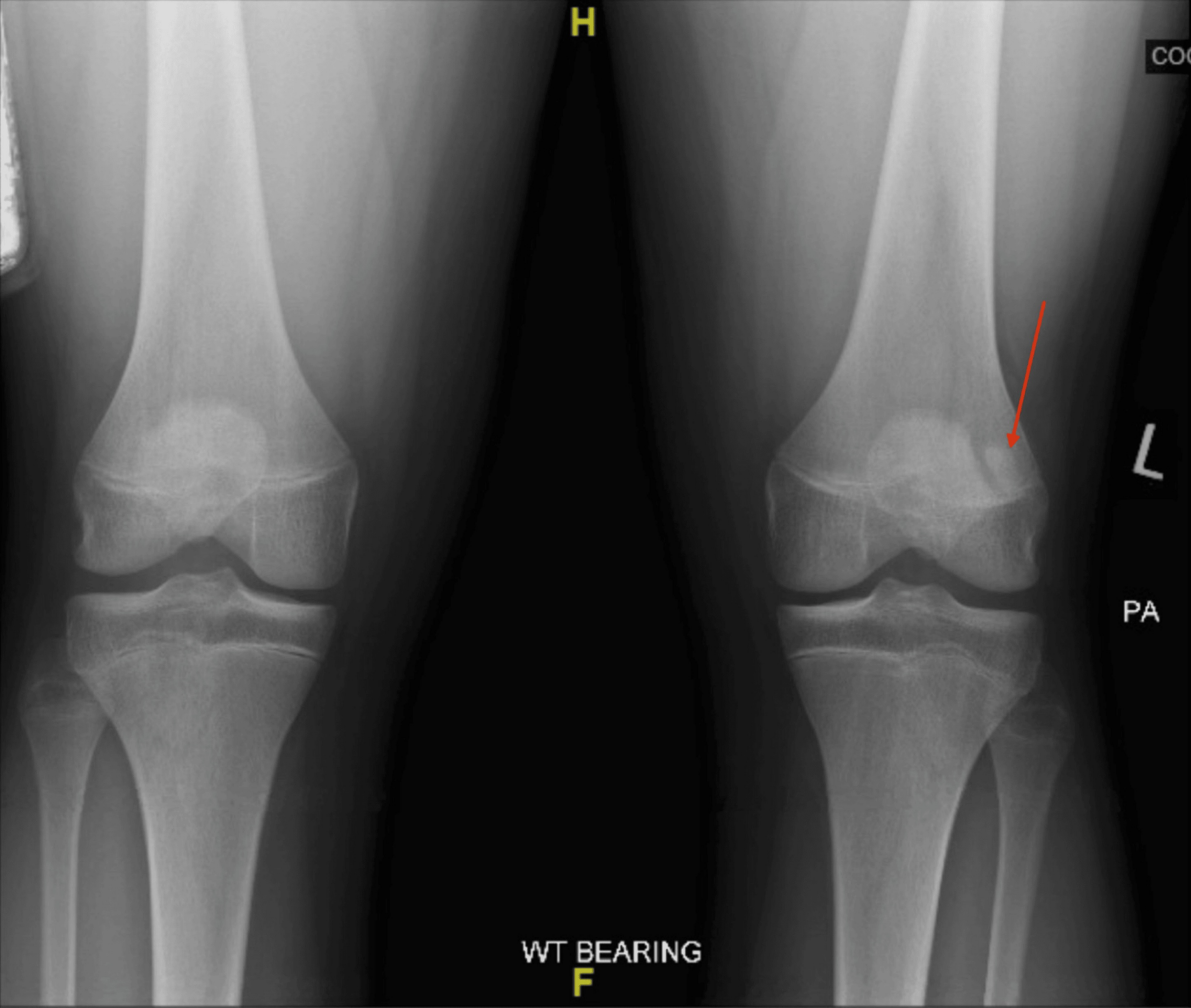
PA weight-bearing film of bilateral knees showing L-sided fragmented patella PA: Posteroanterior.

**Figure 2:**
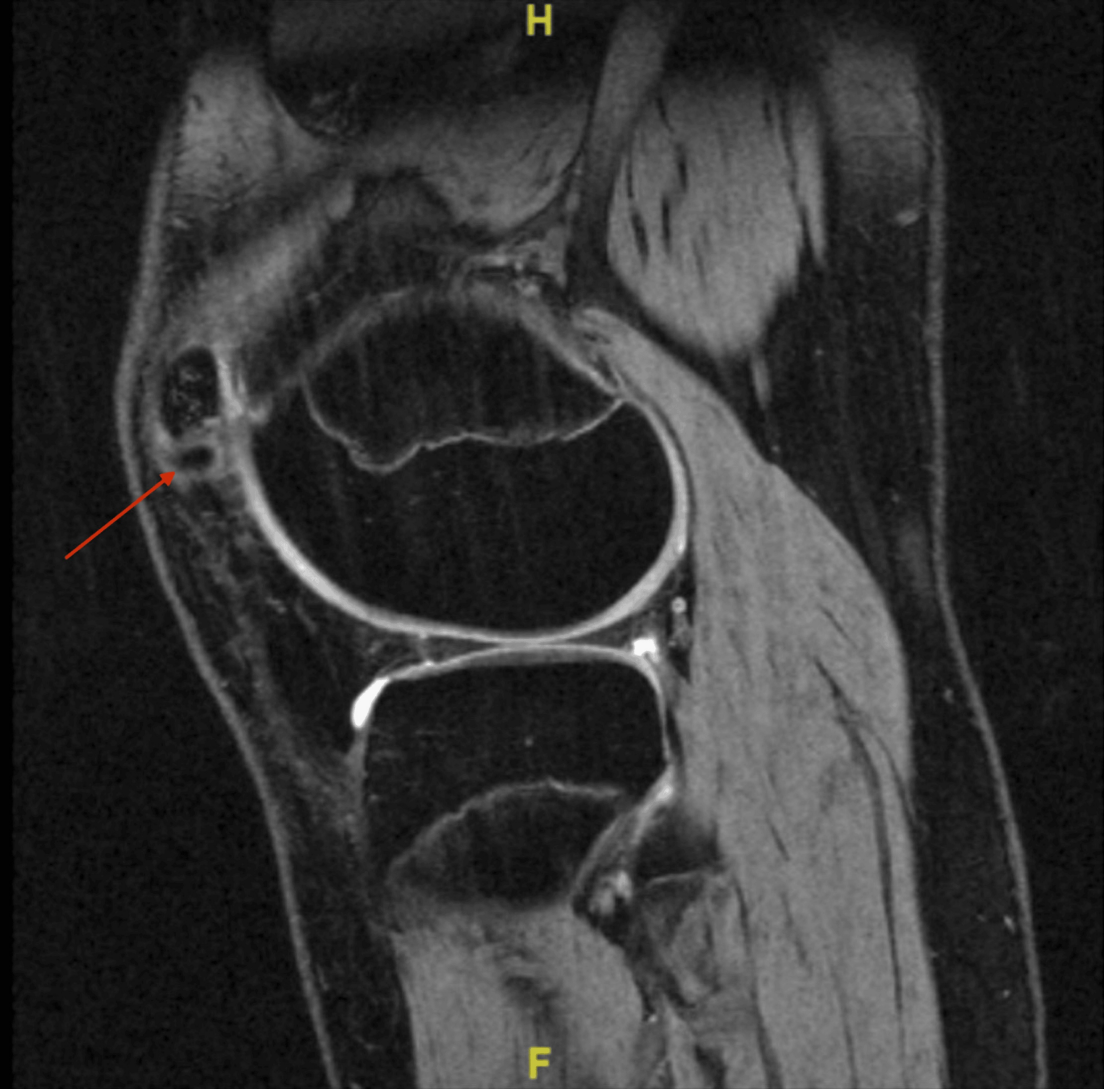
MRI without contrast of L knee showing superolateral portion of bipartite patella

At the time of initial presentation, radiographs of the bilateral knees were obtained (Figure 1). Initially, the left knee radiograph was read by the radiologist as a minimally displaced suprapatellar fracture with associated healing. Initial treatment included conservative therapy with bracing for one month. At the one-month follow-up appointment, the symptoms persisted. Due to the failure of symptomatic improvement, an MRI of the left knee was ordered to evaluate a possible structural issue. The MRI (Figure 2) confirmed that the abnormality observed on the initial radiograph was not the result of a fracture but rather a bipartite patella with surrounding edema, leading to the belief that the synchondrosis of the bipartite patella was disrupted as a result of a traumatic fall.

**Figure 3:**
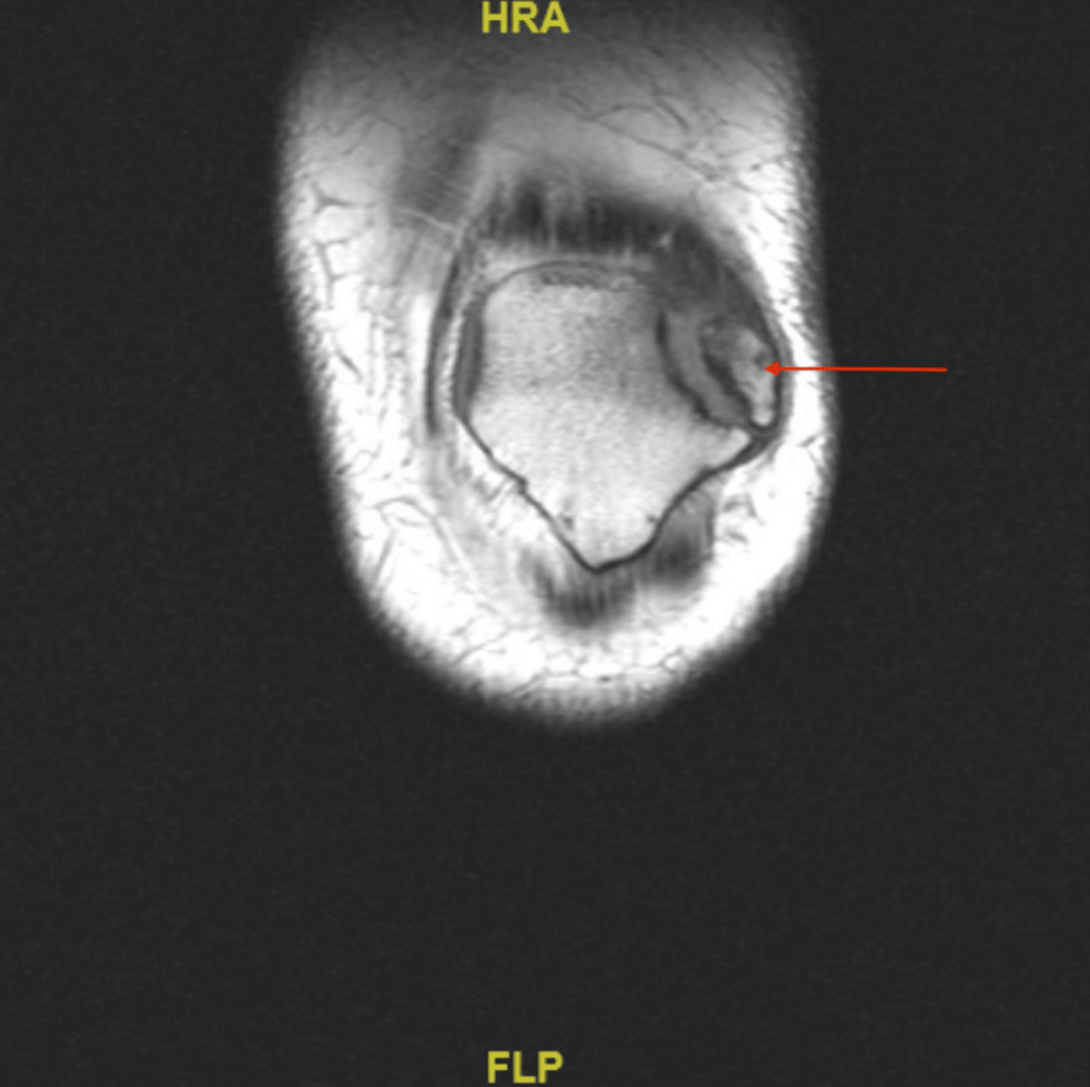
MRI without contrast of L knee showing superolateral portion of bipartite patella with quadriceps tendon attachment

In addition, the MRI of the left knee demonstrated tendinous attachment of the quadriceps musculature to the superolateral accessory bone of the patella (Figure 3). Thus, excision of this portion of bone could result in the patient developing difficulty performing leg extension. Given the risks with excision of the superolateral portion of the bipartite patella, the decision was made to proceed with open reduction and internal fixation (ORIF) of the bipartite patella via arthroscopy.

Surgery was performed with the patient in the supine position under general anesthesia. An anterolateral arthroscopic portal was created to view the intraarticular structures, which revealed no acute abnormalities. The patellofemoral joint was inspected, and any friable tissue was appropriately debrided. The intercondylar notch was visualized to inspect the anterior cruciate ligament (ACL) and posterior cruciate ligament (PCL), both of which were intact. The medial and lateral compartments, along with the gutters, were visualized with no acute abnormalities. Next, the bipartite fragment was identified, and a lateral release was performed with a coblation device. After concluding the arthroscopic portion of the procedure, a straight anterior incision over the superior portion of the patella was made, and dissection was done through cutaneous tissues down to the level of the patella. Fluoroscopic imaging was utilized to identify the precise location of the synchondrosis.

**Figure 4:**
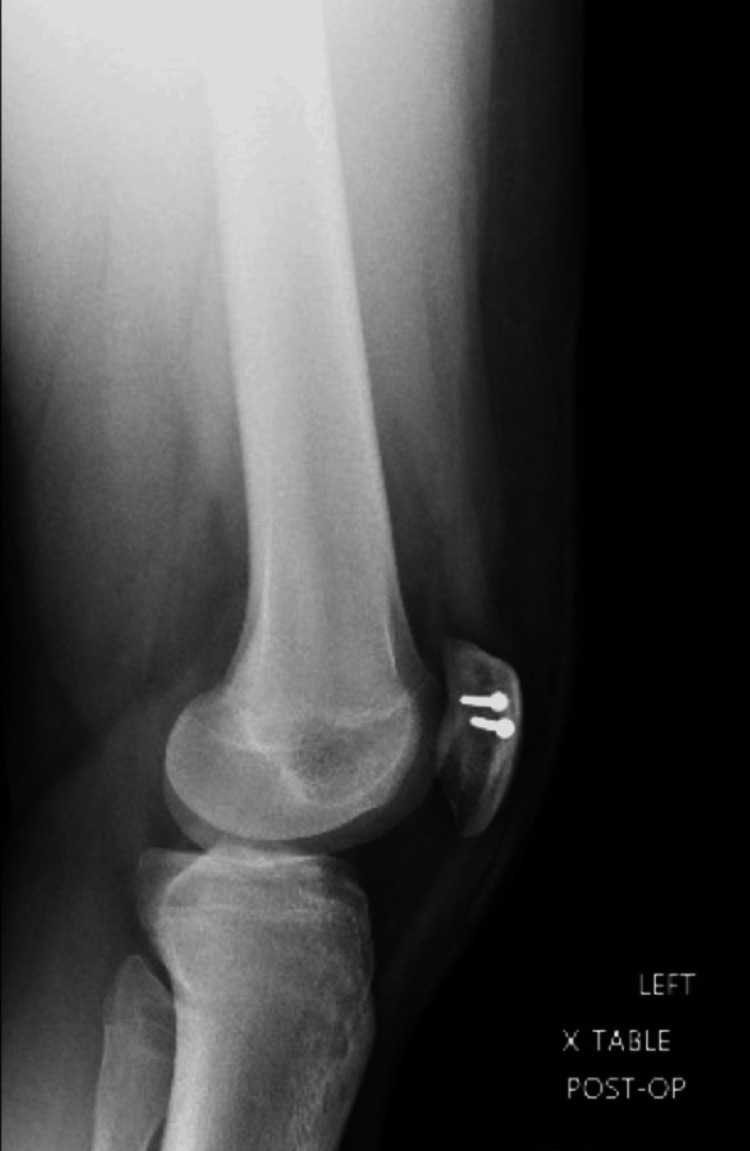
Postoperative X-ray L knee lateral view

**Figure 5:**
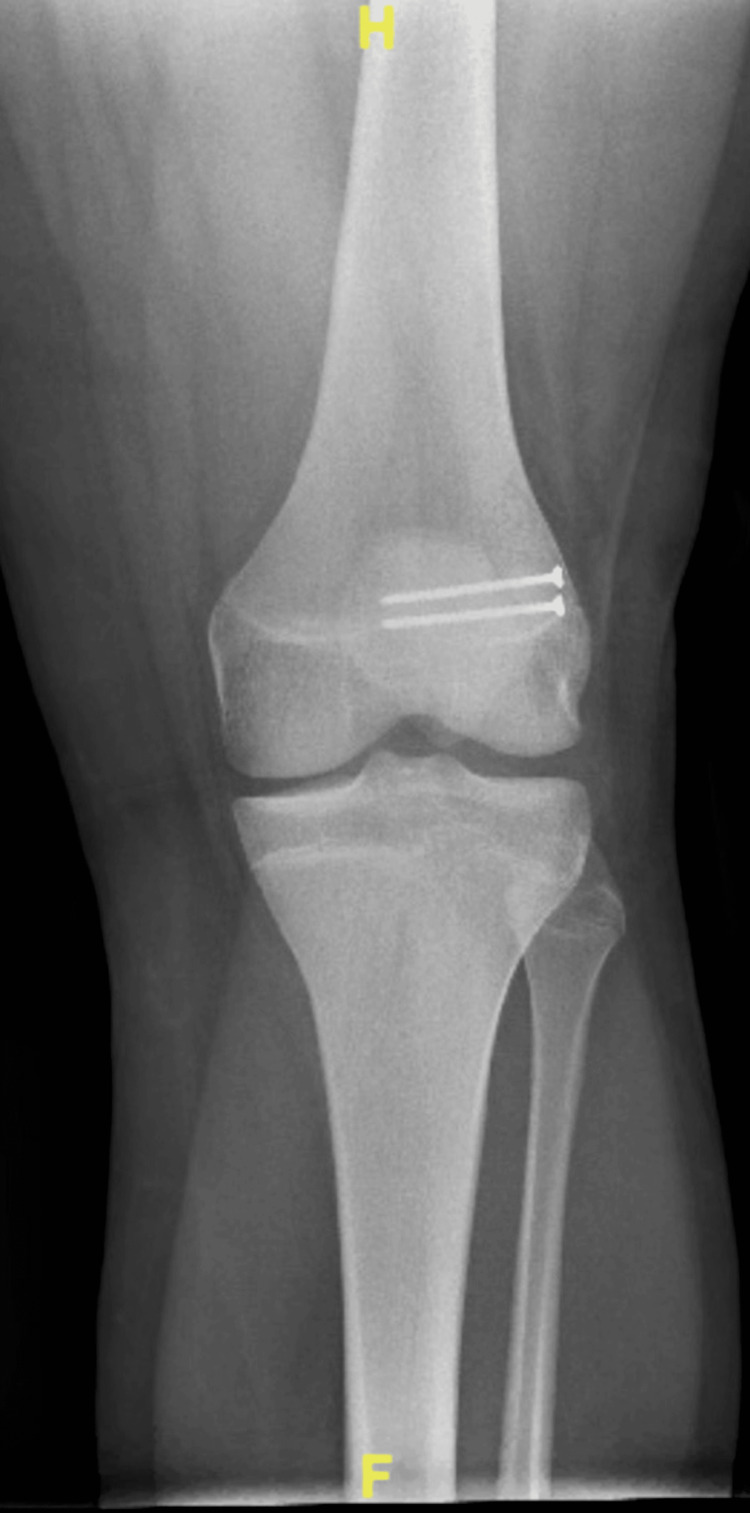
Postoperative X-ray L knee PA view PA: Posteroanterior.

Next, the synchondrosis was dissected with a 15-blade, and a curette was used to scrape any remaining fibrous tissue between the accessory and main patella bones. Once bleeding surfaces were visualized, a reduction clamp was utilized to reduce the bipartite patella as close together as possible. Two full-threaded stainless steel 2.7 mm screws were inserted using a lag technique in a medial-to-lateral fashion perpendicularly to the synchondrosis region to achieve adequate purchase. Knee range of motion demonstrated no restrictions or abnormalities during the arc of motion. Postoperative X-rays (Figures 4, 5) revealed proper placement of screws with a maximum reduction of the bipartite patella. The immediate postoperative plan included placing the knee in a hinged knee brace, weight-bearing as tolerated of the left lower extremity with crutches as needed, oral pain medication as prescribed, and follow-up in two weeks for suture removal and an initial physical therapy visit.

At the patient’s first follow-up appointment (postoperative day 10), he reported minimal pain and swelling. The patient was advised to continue physical therapy, slowly wean off the crutches over the next several weeks, and try to bear weight as tolerated. At the patient’s six-week follow-up, he reported feeling much better with only tenderness at the superomedial patella, full range of motion without crepitus or instability, and well-healed incisions. The patient was instructed to discontinue the use of the hinged knee brace, continue weight-bearing as tolerated, and continue to hold off on major physical activity except for physical therapy and follow-up in six weeks.

**Figure 6:**
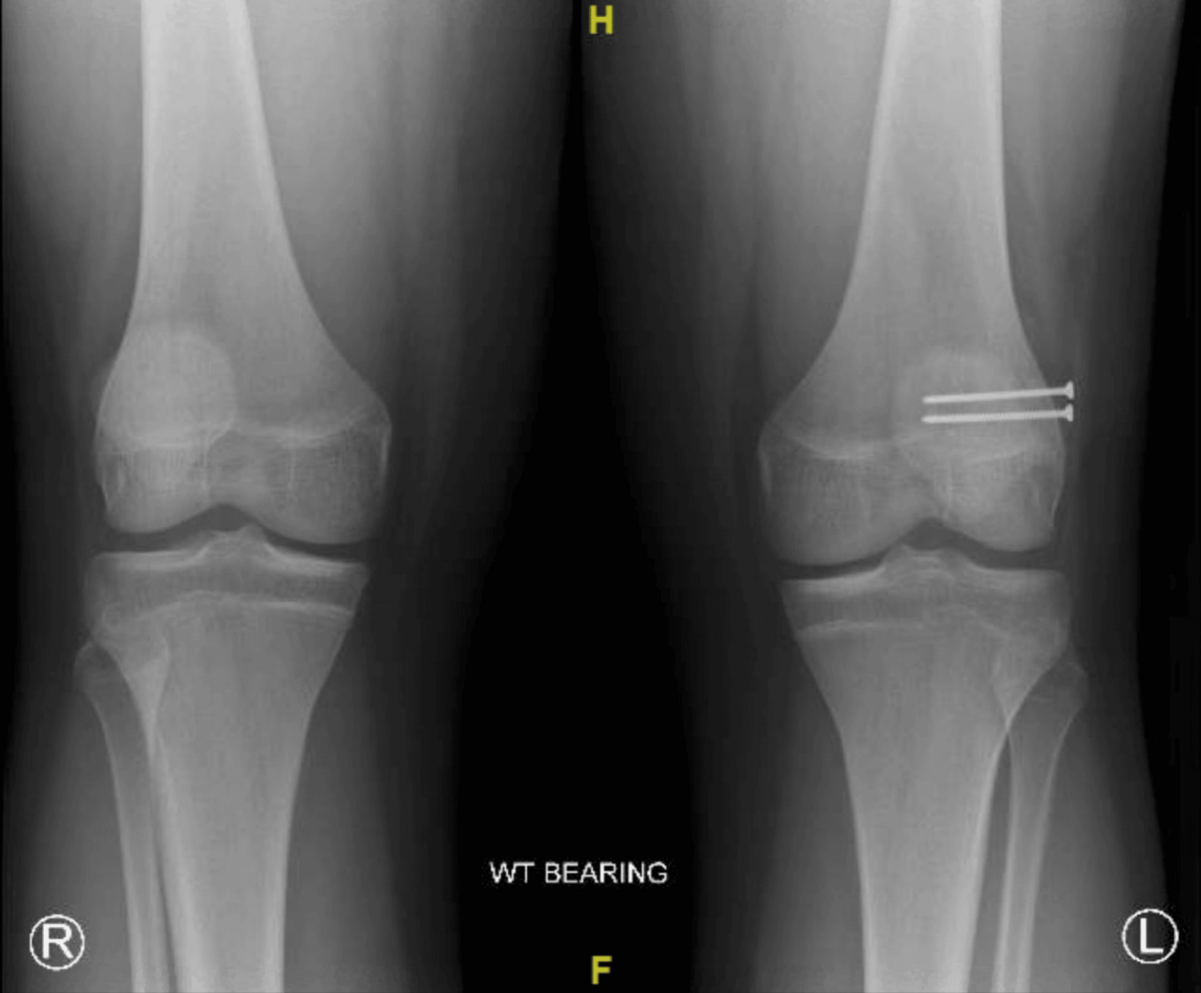
Three-month postoperative X-ray bilateral knee AP weight-bearing view AP: Anteroposterior.

At the subsequent follow-up, the patient reported mild superomedial knee sensitivity but otherwise had no complaints. New radiographs were taken at this visit, which showed implants in a stable position without failure and a decrease in the size of the fibrous synchondrosis when compared to the size before surgery (Figure 6). On physical examination, the left knee was stable with a full range of motion and was without crepitus or effusion. There was mild tenderness to palpation of the superomedial knee. The patient was instructed to continue to increase activities, such as running, as tolerated, to avoid sports for the time being, and to follow up in three months with new radiographs.

**Figure 7:**
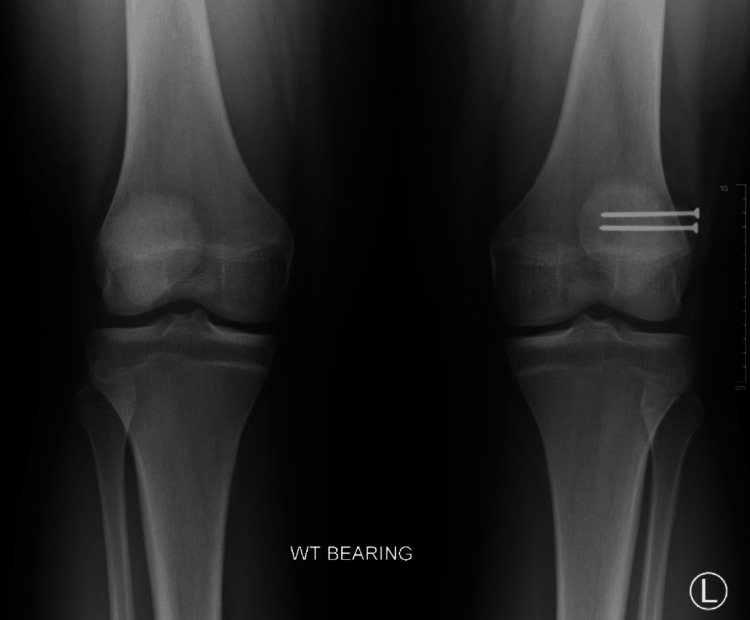
Six-month postoperative X-ray bilateral knee AP weight-bearing view AP: Anteroposterior.

**Figure 8:**
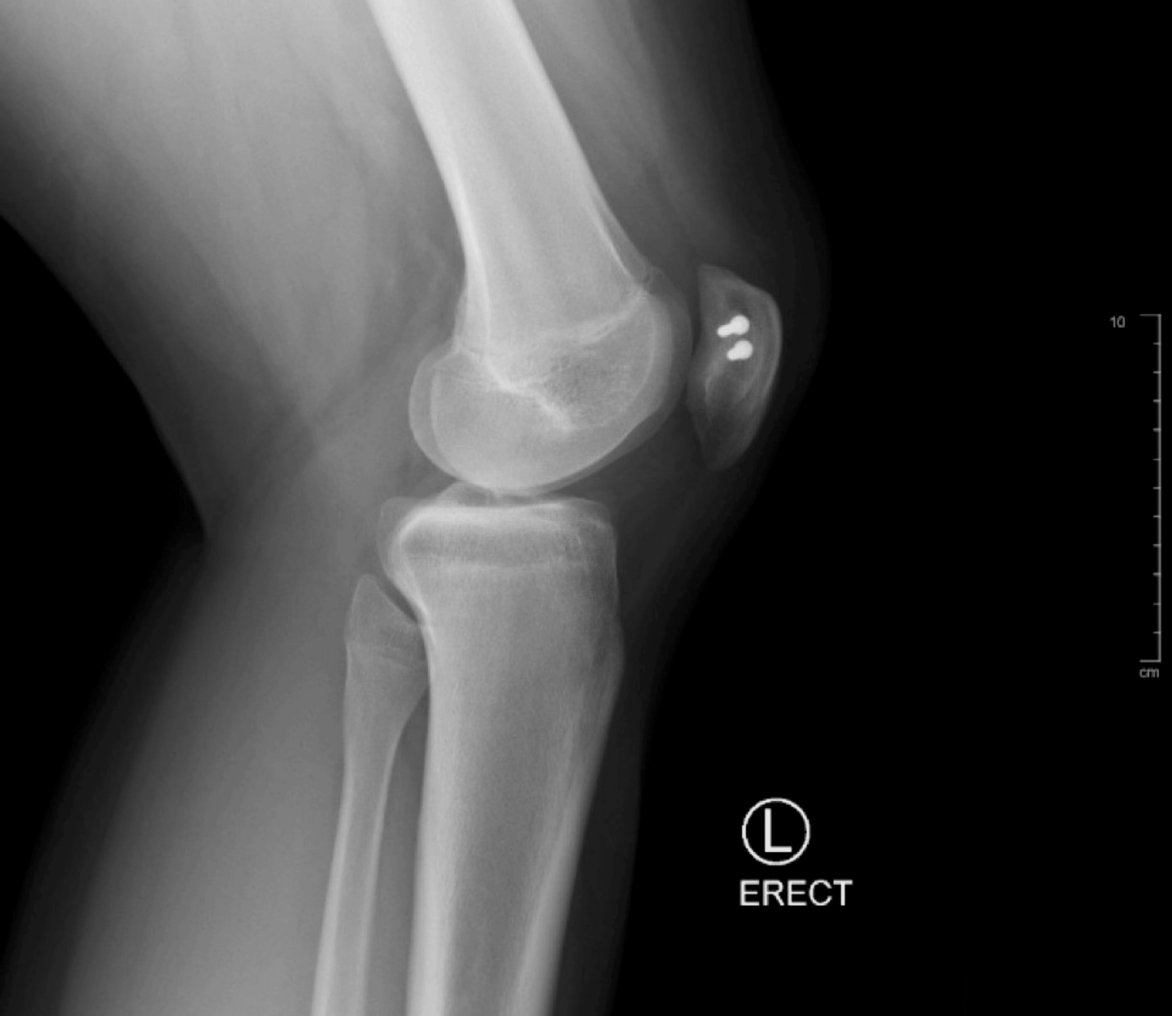
Six-month postoperative X-ray L knee erect view

**Figure 9:**
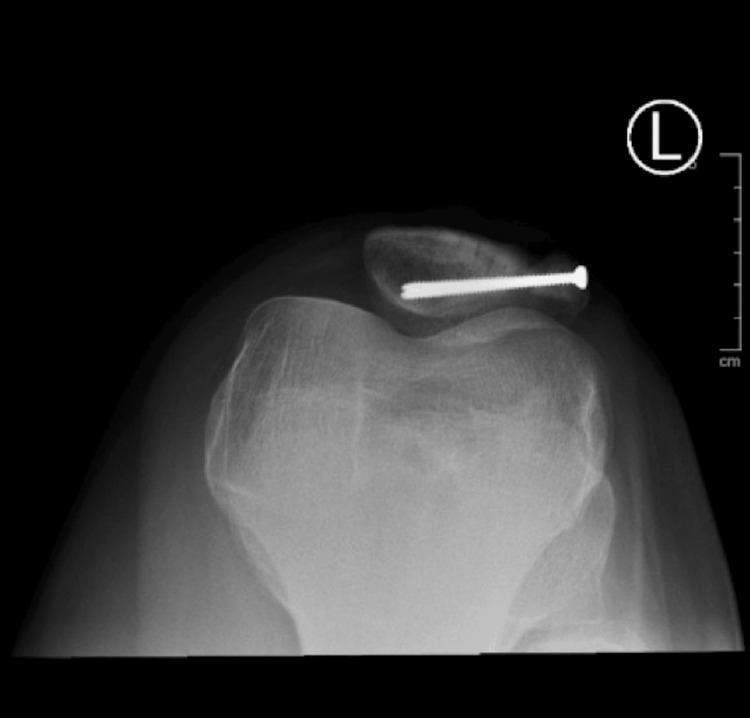
Six-month postoperative X-ray L knee sunrise view

At the patient’s next follow-up, six months post-operation, the patient reported aching pain after working but had overall limited pain and full range of motion without crepitus or instability on physical activity​​. Radiographs showed internal improvement in the synchondrosis with hardware still in the correct positioning (Figures 7, 8, 9). The patient was instructed to continue activities ​​as tolerated with ice and NSAIDs PRN for pain management.

**Figure 10:**
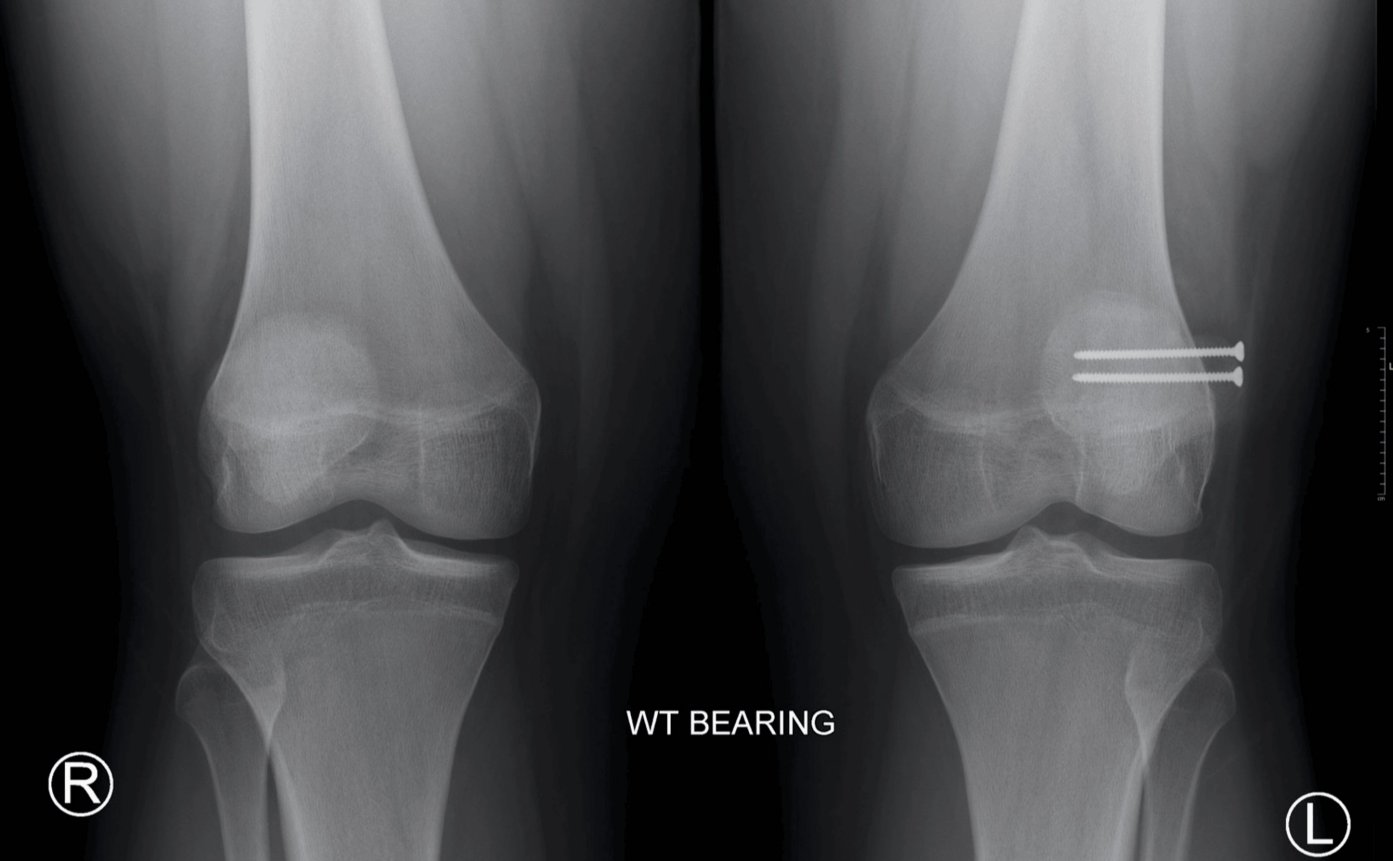
12-month postoperative X-ray B/L knee AP view AP: Anteroposterior; B/L: Bilateral.

**Figure 11:**
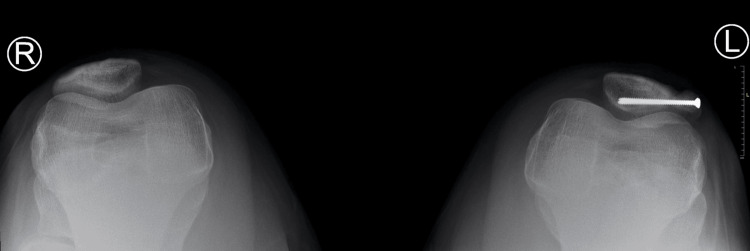
12-month postoperative X-ray B/L knee sunrise view B/L: Bilateral.

At the 12-month follow-up, the patient reported minimal pain with prolonged standing but none with strenuous activity. Repeat radiographs at this visit revealed increased healing of the bipartite portion of the patella with only a small visible lucency at the superior aspect of the patella (Figures 10, 11). The patient was instructed to follow up as needed and to utilize NSAIDs PRN for pain management.

## Discussion

Although many cases of symptomatic bipartite patella resolve with conservative treatment, some patients fail conservative therapy. Atesok et al. noted in their study on treatment alternatives for symptomatic bipartite patella that there should be a minimum of six months of conservative treatment before proceeding to surgery [5]. The authors also mentioned that direct trauma to the patella or impaired activities of daily living, both of which were experienced by the patient in this case report, are justifiable reasons to skip several months of conservative therapy in favor of surgical management. Atesok et al. also mentioned that conservative, outpatient management of this condition is hard to track on a day-to-day basis, leading to issues with compliance.

In a separate systematic review focusing on return to activity in patients with symptomatic bipartite patella, Matic and Flanigan discovered that of 96 cases with documented attempts at conservative therapy, 90 of those patients had surgery to correct the defect [6]. In addition, Matic and Flanigan noted that of the 130 total cases in their review, only 13 patients were able to return to full activities without surgical intervention, raising questions about whether conservative treatment achieves unrestricted return to daily activities and sports.

There are also several surgical options to manage the patient’s pain including ORIF, tension band wiring technique, and excision of accessory bone. Both tension band wiring and excision techniques have their advantages and disadvantages. Both have the advantage that they could be performed arthroscopically or in an open technique [7]. Tension band wiring, which involves threading a wire through the quadriceps tendon to achieve proper compression after the reduction of a patellar fracture, can lead to good fracture healing and union but can lead to superficial pain and hardware removal [8-10].Surgical excision of the accessory fragment through an open or arthroscopic approach is also well-tolerated [4,11-13].

Of the techniques mentioned, open surgical excision is the most common, but that does not mean it is without disadvantages. Matic and Flanigan noted in their review that 67% of the patients who underwent surgery to correct symptomatic bipartite patella had surgical excision, but almost 10% of these patients had residual symptoms.They also reported that while open surgical excision is more common than arthroscopic excision, it leads to increased quadriceps weakness, larger joint effusions, and an overall longer recovery period compared to arthroscopically managed patients.

Ishikawa et al. also reported similar effects, as six patients who had undergone open excision required drainage of a large joint effusion within two weeks of the operation [14]. Another limitation of the open surgical excision is that if the fragment of the accessory patella is large enough, removal can lead to a subsequent patellar incongruity and eventually could lead to patellofemoral arthritis. In this circumstance, which was encountered in the patient in this case report, ORIF was the best option as it maintained the normal anatomy intact, ostensibly preventing any instability or incongruities. Although most cases of symptomatic bipartite patella resolve either spontaneously or after conservative treatments, sometimes surgical intervention may be indicated. Overall, most surgical techniques lead to the union of the two segments of the patella along with few complications.

## Conclusions

Bipartite patella syndrome is usually an asymptomatic, coincidental finding in adolescents but can sometimes cause anterior knee pain. While most cases resolve spontaneously or with conservative treatment, some patients require surgical correction of the patella. Typically, surgical intervention involves excising the accessory fragment. However, if the fragment is too large or has a tendinous attachment to the accessory fragment, ORIF is a more appropriate alternative. Overall, the goal of ORIF is to achieve a union between the two segments of the patella, but this comes with a few complications. However, if a case progresses to the need for surgical intervention, then providers need to assess the risk-benefit analysis for each type of procedure to prevent the fewest complications and the most complete resolution of symptoms.

## References

[1] McMahon SE, LeRoux JA, Smith TO, Hing CB (2016). The management of the painful bipartite patella: a systematic review. Knee Surg Sports Traumatol Arthrosc.

[2] Canizares GH, Selesnick FH (2003). Bipartite patella fracture. Arthroscopy.

[3] Saupe E (1921). Beitragzur patella bipartita. Fortschr Rontgenstr.

[4] Ireland ML, Chang JL (1995). Acute fracture bipartite patella: case report and literature review. Med Sci Sports Exerc.

[5] Atesok K, Doral MN, Lowe J, Finsterbush A (2008). Symptomatic bipartite patella: treatment alternatives. J Am Acad Orthop Surg.

[6] Matic GT, Flanigan DC (2015). Return to activity among athletes with a symptomatic bipartite patella: a systematic review. Knee.

[7] Werner S, Durkan M, Jones J, Quilici S, Crawford D (2013). Symptomatic bipartite patella: three subtypes, three representative cases. J Knee Surg.

[8] Okuno H, Sugita T, Kawamata T, Ohnuma M, Yamada N, Yoshizumi Y (2004). Traumatic separation of a type I bipartite patella: a report of four knees. Clin Orthop Relat Res.

[9] Peek AC, Barry M (2012). Patella fracture in a boy with bilateral inferior pole bipartite patellae. Knee.

[10] Tauber M, Matis N, Resch H (2007). Traumatic separation of an uncommon bipartite patella type: a case report. Knee Surg Sports Traumatol Arthrosc.

[11] Halpern AA, Hewitt O (1978). Painful medial bipartite patellae: a case report. Clin Orthop Relat Res.

[12] Ishikawa H, Sakurai A, Hirata S, Ohno O, Kita K, Sato T, Kashiwagi D (1994). Painful bipartite patella in young athletes. The diagnostic value of skyline views in squatting position and surgical excision. Clin Orthop Relat Res.

[13] Bourne MH, Bianco AJ Jr (1990). Bipartite patella in the adolescent: results of surgical excision. J Pediatr Orthop.

[14] Ishikawa H, Sakurai A, Hirata S, Ohno O, Kita K, Sato T, Kashiwagi D (1994). Painful bipartite patella in young athletes. The diagnostic value of skyline views taken in squatting position and the results of surgical excision. Clin Orthop Relat Res.

